# Multilocus characterization and phylogenetic analysis of *Leishmania siamensis* isolated from autochthonous visceral leishmaniasis cases, southern Thailand

**DOI:** 10.1186/1471-2180-13-60

**Published:** 2013-03-18

**Authors:** Saovanee Leelayoova, Suradej Siripattanapipong, Atitaya Hitakarun, Hirotomo Kato, Peerapan Tan-ariya, Padet Siriyasatien, Seksit Osatakul, Mathirut Mungthin

**Affiliations:** 1 Department of Parasitology, Phramongkutklao College of Medicine, Bangkok 10400, Thailand; 2 Department of Microbiology, Faculty of Science, Mahidol University, Bangkok 10400, Thailand; 3 Department of Disease Control, Laboratory of Parasitology, Graduate School of Veterinary Medicine, Hokkaido University, Sapporo 060-0818, Japan; 4 Department of Parasitology, Faculty of Medicine, Chulalongkorn University, Bangkok 10330, Thailand; 5 Department of Pediatrics, Faculty of Medicine, Prince of Songkla University, Songkhla 90110, Thailand

**Keywords:** *Leishmania siamensis*, Phylogenetic analysis, Multilocus characterization, Thailand, Lineage

## Abstract

**Background:**

Visceral leishmaniasis (VL) caused by *Leishmania siamensis* is an emerging disease continuously reported in six southern provinces of Thailand. To date, the phylogenetic relationships among *Leishmania* isolates from Thai patients and other *Leishmania* species are still unclear and the taxonomic diversity needs to be established. In this study, the phylogenetic inference trees were constructed based on four genetic loci (i.e., SSU-rRNA, ITS1, *hsp*70, and *cyt* b), using DNA sequences obtained from autochthonous VL patients from southern Thailand and reference sequences of reported *Leishmania* isolates from other studies deposited in GenBank.

**Results:**

Phylogenetic analyses of *hsp*70 and *cyt* b loci supported a clade comprised of *L. siamensis* isolates, which is independent to the other members in the genus *Leishmania*. In combination with genetic distance analysis, sequence polymorphisms were observed among *L. siamensis* isolates and two different lineages could be differentiated, lineages PG and TR. Phylogenetic analysis of the *cyt* b gene further showed that *L. siamensis* lineage TR is closely related to *L. enrietti*, a parasite of guinea pigs.

**Conclusion:**

The finding of this study sheds further light on the relationships of *L. siamensis*, both in intra- and inter-species aspects. This information would be useful for further in-depth studies on the biological properties of this important parasite.

## Background

Leishmaniasis, one of the most important neglected infectious diseases, is endemic in 88 tropical and subtropical countries. In the past, Thailand was thought to be free of leishmaniasis. From 1960–1986, sporadic cases were reported among Thais who had visited the endemic areas [1-3]. Since then, a few autochthonous cases of leishmaniasis caused by *L. infantum* and *L. donovani* were reported in 1996, 2005 and 2007; however, the sources of infection were not identified [4-6]. In 2008, based on sequence comparison of two genetic loci, *Leishmania siamensis*, a novel species causing autochthonous leishmaniasis (VL), was described for the first time in a Thai patient from a southern province of Thailand [7]. The analysis of three protein-coding genes revealed that the taxonomic position of *L. siamensis* is closely related to *L. enrietti*, a *Leishmania* of guinea pigs [8]. To date, more than ten autochthonous VL cases caused by *L. siamensis* were sporadically reported in six southern, one eastern and three northern provinces of Thailand [8,9]. Due to the continually increasing number of cases, it is speculated that subclinical and clinical leishmaniasis in Thailand might exist in high numbers which needs prompt diagnosis.

The sequences of various genetic markers have been used to study the parasite diversity and relationships within *Leishmania* including the sequences of DNA polymerase α [10], RNA polymerase II [10], 7SL RNA [11], ribosomal internal transcribed spacer [12-14], the N-acetylglucosamine-1-phosphate transferase gene [15], mitochondrial cytochrome b gene [16] and heat shock protein 70 gene [17]. Building a database of sequences of new local isolates of *Leishmania* in Thailand, together with the published *Leishmania* sequences from GenBank, could be useful for future comparison studies. Therefore, this study aimed to genetically characterize *L. siamensis* isolated from five Thai VL patients, based on four genetic loci, i.e., small subunit ribosomal RNA (SSU-rRNA), internal transcribed spacer 1 (ITS1) region, heat shock protein 70 (*hsp*70), and cytochrome b (*cyt* b). In addition, we studied the phylogenetic relationships of *L. siamensis* within the genus *Leishmania* by comparison with retrieved sequences of other *Leishmania* species from GenBank.

## Methods

### *Leishmania* from VL Thai patients

Samples used in this study were collected from five autochthonous VL patients reported from Phang-nga, Trang, Songkla, and Stun provinces, southern Thailand. All patients presented with hepatosplenomegaly and pancytopenia. Amastigotes were identified under microscope from Giemsa-stained bone marrow smears in all cases. Two axenic cultures of promastigotes were obtained using bone marrow aspirates in Schneider’s medium supplemented with 20% FBS. Genotypic characterization was processed on three positive clinical samples (i.e., Giemsa-stained bone marrow smears and buffy coat) and two cultured promastigotes. The information of these samples is shown in Table 1.

**Table 1:** The characteristics of five samples of autochthonous leishmaniasis used in this study

**Isolates**	**Location**	**Year of isolation**	**Clinical presentation of leishmaniasis**	**HIV coinfection**	**Source of DNA**	**Sequence accession no. [reference]**			
						**SSU-rRNA**	**ITS1**	***hsp*** **70**	***cyt *** **b**
CU1	Songkhla	2011	VL^#^	Yes	Culture	JX195633	JX195639	KC202883	JX195635
PCM1^+^	Phang-nga	2007	VL	Yes	Bone marrow smear	JN885899 [8]	EF200012 [7]	not sequenced	JX195636
PCM2^§^	Trang	2010	CL^*^ and VL	Yes	Culture	JQ280883 [8]	JX195640	KC202880	JX195634
PCM4	Stun	2010	VL	No	Bone marrow smear	JN087497	JX195637	KC202882	not sequenced
PCM5	Trang	2011	CL and VL	Yes	Buffy coat	not sequenced	not sequenced	KC202881	not sequenced

^+^, this isolate was previously described in the study by Sukmee et al. [7]; ^§^, this isolate was previously described as Trang strain in the study by Bualert et al. [8]; ^#^, visceral leishmaniasis; ^*^, cutaneous leishmaniasis.

### Ethics statement

The study was approved by the Ethics Committee of the Royal Thai Army Medical Department, Thailand. No information on the patients was presented in this study.

### DNA preparation

DNA was extracted from the Giemsa-stained smears of bone marrow using modified FTA extraction paper (Whatman, Bioscience, USA) following the protocol as previously described [18]. The Genomic DNA Mini Kit (Tissue) (Geneaid, USA) was used to extract the DNA from other three remaining samples.

### PCR amplification

PCR assays were used to amplify a fragment of four genetic loci using the previously described conditions, i.e., SSU-rRNA [19], ITS1 region [20], *hsp*70 [21], and *cyt* b [22]. The PCR products were subjected to electrophoresis on 1.5% agarose gels and stained with SYBR safe (Invitrogen, USA). Gels were photographed and documented on high-density printing paper using Uvisave gel documentation system I (Uvitech, UK).

### Cloning and sequencing

PCR products amplified from the four loci were purified using a Wizard® SV Gel and PCR Clean-Up System (Promega, Madison, USA) according to the manufacturer instructions and then directly sequenced. For the PCR products that had insufficient amounts of DNA for direct sequencing, they were cloned in *E. coli* competent cells to produce a higher quantity of identical DNA. For cloning, the purified PCR product was ligated into pGEM-T Easy vector (Promega, Madison, USA). The ligated product was introduced into the *E. coli* strain JM109 by chemical transformation. One colony from each cloning reaction was selected. The recombinant plasmids were purified using Wizard® Plus SV Minipreps DNA purification system (Promega, Madison, USA) and bidirectional sequenced using universal primer T7 and SP6. DNA sequencing was conducted by 1^st^ Base Pte. Ltd., Singapore. The chromatograms were validated and assembled in BioEdit version 7.0.1.

### Phylogenetic analysis

The sequences were multiple-aligned with a set of *Leishmania* strains retrieved from the GenBank using ClustalX, version 2.0.12 [23]. The pairwise genetic distances among isolates were estimated using program MEGA (Molecular Evolutionary Genetics Analysis), version 4.0 [24]. To investigate the relationships among *L. siamensis* isolates and other *Leishmania* species, *Leishmania* sequences of each locus examined in this study from GenBank were included in the dataset. The evolutionary history was inferred by phylogenetic tree construction using three methods, i.e., Neighbor Joining (NJ), Maximum Parsimony (MP) and Bayesian inference. The NJ and MP trees were constructed using program MEGA, version 4.0 [24]. Reliability of the inferred trees was tested by 1000 bootstrap replications. For the Bayesian method, starting trees were random: four simultaneous Markov chains were run for 500,000 generations, burn-in values were set at 30,000 generations and trees were sampled every 100 generations. Bayesian posterior probabilities were calculated using a Markov Chain Monte Carlo sampling approach implemented in MrBAYES, version 3.1.2 [25]. The Akaike information criterion in Modeltest, version 3.06, was used to select a DNA substitution model of all phylogenetic analyses [26]. The following models were selected for the dataset of each gene: K2P (SSU-rRNA), TrN+Γ (ITS1 and *hsp*70), and GTR+Γ (*cyt* b). The nucleotide sequences generated in this study have been deposited in GenBank under accession no. JX195633-JX195637, JX195639-JX195640, and KC202880-KC202883.

## Results

### Sequence analysis

PCR amplification of each target locus resulted in amplicons of the expected sizes as follows: SSU-rRNA (540 bp), ITS1 (340–348 bp), *hsp*70 (1422 bp), and *cyt* b (865 bp). Due to the limited amount of DNA samples, studied loci of some samples were not successfully amplified. Twelve amplicons were successfully amplified and bidirectionally sequenced. As a result, a total of 15*L. siamensis* sequences were analyzed in this study. These consisted of four isolates from SSU-rRNA (CU1, PCM1, PCM2, and PCM4; sequences of PCM1 and PCM2 were reported by Bualert et al. [8]), four isolates from ITS1 (CU1, PCM1, PCM2, and sequences of PCM4; PCM1 were reported by Sukmee et al. [7]), four isolates from *hsp*70 (CU1, PCM2, PCM4, and PCM5), and three isolates from *cyt* b (CU1, PCM1, and PCM2).

The comparison of mean genetic distance among the five isolates of each respective target was not calculated since different sets of isolates were used for each marker (Table 1). However, our preliminary analysis using available *L. siamensis* isolates indicates that the overall mean genetic distance varied depending on the markers analyzed. The most variable marker was the ITS1 region, followed by the *cyt* b gene, and the *hsp*70 gene whereas the SSU-rRNA sequences were identical for all isolates.

Sequence analysis could divide the *L. siamensis* isolates into two groups; the first one consisted of four isolates (isolates CU1, PCM1, PCM4, and PCM5), and the second group consisted of only one isolate (isolate PCM2).

According to these results, the isolates of groups 1 and 2 could be considered as different lineages and primarily designated as lineages PG (isolates CU1, PCM1, PCM4, and PCM5) and TR (isolate PCM2), respectively. In addition, the genetic divergence between TR and PG lineages was much higher than usually observed within other species (data not shown).

### Phylogenetic analysis

Three phylogenetic analyses using the NJ, MP, and Bayesian methods were performed to observe the relationships between two *L. siamensis* lineages. Using three different constructing methods, the trees showed similar phylogenetic topology for all four loci supported by related bootstrapping/posterior probability values.

**Figure 1:**
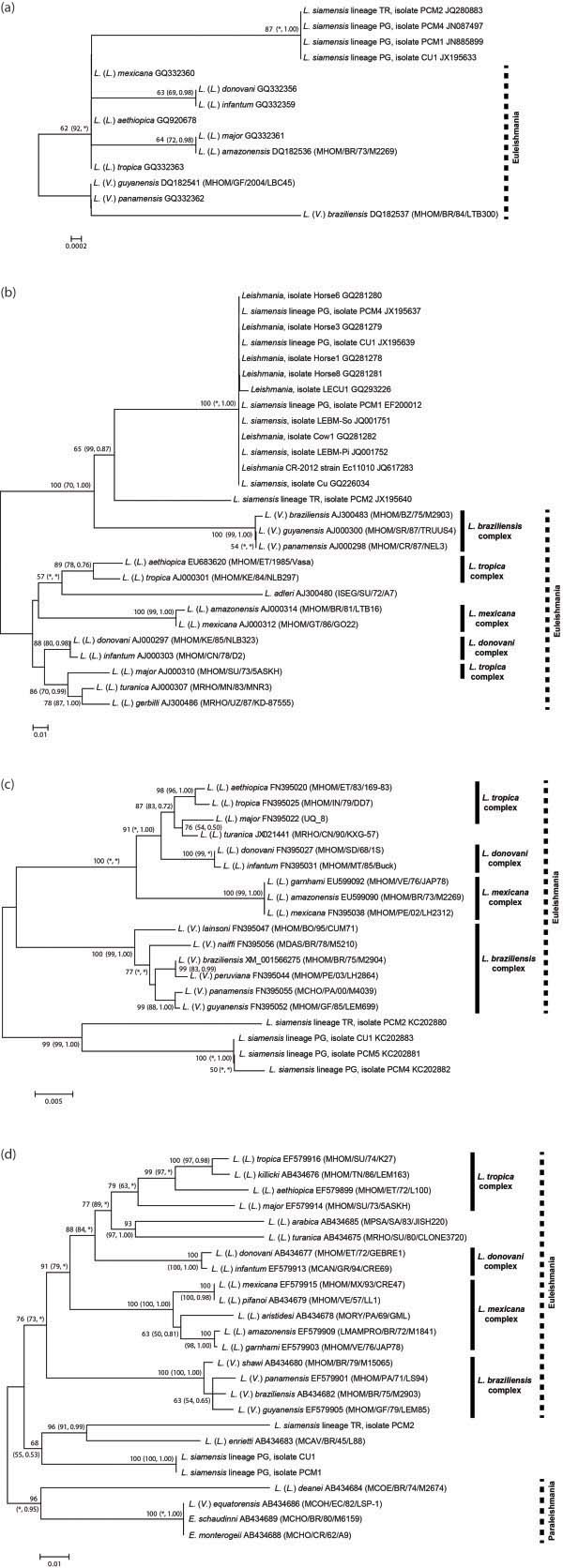
**The unrooted phylogenetic tree inferred from DNA sequences of four markers using Neighbor Joining method.** The bootstrapping values less than 50 are omitted. The bootstrapping and posterior probability values estimated by Maximum parsimony and Bayesian inference methods are shown in parenthesis at each node, respectively. Asterisks indicate bootstrapping and posterior probability values that are below 50 or 0.5 or are not calculated by the analyses. Dense lines indicate *Leishmania* species complexes as described by Lainson and Shaw [30]. The species complex of *L. adleri*, *L. turanica*, *L. gerbilli*, and *L. arabica* are unclassified. Dot lines indicate the lineage sections suggested by Cupolillo et al. [35]. (**a**) SSU-rRNA, (**b**) ITS1, (**c**) *hsp*70, (**d**) *cyt* b.

Regarding the phylogenetic tree inferred from each locus, the SSU-rRNA tree was constructed using four *L. siamensis* isolates and ten reference sequences of different *Leishmania* species (Figure 1a). The phylogenetic analyses grouped both *L. siamensis* lineages PG and TR together in a separated clade apart from other *Leishmania* species. Although lineages PG and TR were closely related according to the SSU-rRNA analysis, these two lineages formed separate clades in the phylogenetic tree inferred from other three markers. The ITS1 analysis of 13 *Leishmania* reference sequences and 14 *L. siamensis* sequences revealed a close relationship of *L. siamensis* to the members of *L. braziliensis* complex by forming a strongly supported cluster with both lineages PG and TR. Moreover, *L. siamensis* lineage TR formed a separate branch from the lineage PG but still shared a close relationship (Figure 1b). Interestingly, *L. siamensis* lineage PG clustered with the reference sequences previously isolated from Thai patients (GQ226034, GQ293226, JQ001751, and JQ001752), horse (JQ617283) in USA, and those isolated from a cow (CQ281282) and horses (CQ281278, CQ281279, CQ281280, and CQ281281) in Europe. Among these isolates, 100% sequence identity was revealed, except 99.6% identity of the isolate LECU1. For the *hsp*70 region, the phylogenetic tree was constructed using 15 reference sequences and four *L. siamensis* sequences. Both *L. siamensis* lineages apparently formed independent monophyletic clades outside the clusters of those other species while each *L. siamensis* lineage was still separated into different branches (Figure 1c). In addition, constructing the *cyt* b tree using 20 reference *Leishmania* sequences, two *Endotrypanum* sequences, and three *L. siamensis* sequences, similar results were observed. This tree showed high congruence to *hsp*70 tree since all taxa were concordantly clustered into the same species complex and placed *L. siamensis* at the basal branch of *Leishmania* in Euleishmania section.

In addition, the *L. siamensis* lineage TR was closely related to *L. enrietti* whereas lineage PG was furcated into a sister clade (Figure 1d). For sequence alignments of the ITS1, *hsp*70, and *cyt* b regions, see Additional files 1, 2, 3.

## Discussion

This study characterized *L. siamensis* isolated from autochthonous VL Thai patients based on sequencing of four genetic loci. The construction of molecular evolutionary trees of *Leishmania* species has been extensively studied on various genetic markers both in conserved and variable regions [10-17]. The results of these studies allow us to view evolutionary processes, classify and discriminate species among *Leishmania* isolates. One of the widely used genetic markers for phylogenetic studies is the ribosomal RNA gene. This gene has proved to be useful for inferring the relationships of a wide range of organisms, including *Leishmania*[7,27]. Even though the phylogenetic study based on the complete SSU-rRNA has shown that the variation of this gene limits the classification of this parasite at the subgenus level, studying the phylogenetic position using this gene is fundamentally required for a novel species, like *L. siamensis*[28,29]. In this study, *L. siamensis* was grouped in the monophyletic branch of subgenus *Leishmania* (*Leishmania*) at a long distance in a unique subclade, primarily suggesting that this novel species is closely related to the members of *L.* (*Leishmania*) but evolved rapidly and nonrelative to the members in this subgenus. The incapability to discriminate between two lineages of *L. siamensis* proposed from the genetic distance analysis was not beyond our expectation since the studied region of this gene was remarkably conserved. Unfortunately, the sequences of the Paraleishmania section could not be included in this tree because the SSU-rRNA fragment amplified in this study is located in a different region than those deposited in the GenBank.

Recently, Asato et al. [16] and Fraga et al. [17] analyzed the phylogeny of genus *Leishmania* using the sequences obtained from the *cyt* b and the *hsp*70 regions and demonstrated the improvement of *Leishmania* classification from the traditional method proposed by Lainson and Shaw [30]. Their studies showed that these genes contained sufficient information for distinguishing species/subspecies and also human/nonhuman *Leishmania*. The high congruency between the *cyt* b and the *hsp*70 trees corresponding to the current classification were, thus, logically acceptable as the precise relationship of genus *Leishmania*. Employing the *L. siamensis* taxa into these trees provided more knowledge of this species in relation to other previously identified *Leishmania* species. Previous studies showed the early divergence of *L. enrietti* from other *Leishmania* groups, closely related to genus *Endotrypanum*, suggesting that this species may not belong to genus *Leishmania *[16]. In this study, grouping between both lineages of *L. siamensis* and *L. enrietti* rearranged the phylogenetic position of *L. enrietti* compared with a previous tree shown by Asato et al. [16]. The close relationship between lineage TR (previously described as Trang strain) and *L. enrietti* was supported by our previous work using concatenated sequences of three *Leishmania* protein-coding genes to construct the tree [8]. As shown in this study, *L. enrietti* and *L. siamensis* formed independent sister clades and shared the same branch of the members classified as Euleishmania, leaving the group of Paraleishmania completely separated. This finding distinctly indicated that they might be part of an unclassified subgenus of *Leishmania*. Unfortunately, the *hsp*70 sequences of *L. enrietti* and other species belonging to Paraleishmania were not available in the GenBank, and the alternative notion of this idea could not be obtained by the *hsp*70 tree in this study. However, the phylogenetic position of *L. siamensis* was in good agreement between the *hsp*70 and the *cyt* b trees in that these species were members of neither *L.* (*Leishmania*) nor *L.* (*Viannia*) and they should be regarded as an unclassified subgenus.

Since the identification of *L. siamensis* from a Thai VL case has been described using the comparison of mini-exon and ITS1 sequences in 2008 [7], more cases presumably caused by the same *Leishmania* species were reported on other continents. In 2009, autochthonous cutaneous leishmaniasis (CL) was reported in horses and a cow in Switzerland and Germany, followed by an additional case in a mare from the USA in 2012 [31-33]. These cases showed high ITS1 similarity compared with those previous reports of *L. siamensis*. To elucidate the relationship among the *Leishmania* detected from these cases and *L. siamensis*, these sequences were phylogenetically analyzed. The phylogenetic tree of ITS1 region, again, separated the *L. siamensis* lineage TR from lineage PG. The autochthonous *Leishmania* cases in livestock, previously reported in Europe and the USA, were closely related to the *L. siamensis* lineage PG, suggesting that lineage PG might not be indigenous. Although the relationship of these isolates was strongly supported by the posterior probability/bootstrapping values and nucleotide identity (99-100%), the studies on the isolates from Europe and the USA were limited only on the ITS1 region [31,32]. Thus, the conclusion that the isolates from Thailand and other geographic areas share the same lineage is still premature. Further studies are needed to explore naturally infected reservoir animals like those found in Europe and the USA. More data of their biology, pathology and molecular biology as well as the transmission vectors are required before making conclusions about the relationship of *Leishmania* from these three different geographical areas.

Regarding the phylogenetic trees constructed in this study, the relationships between *L. siamensis* and other *Leishmania* species of SSU-rRNA and ITS1 apparently revealed conflicting phylogenetic signals to the other two markers examined in this study. These could be explained by the different evolutionary constraints displayed by each independent gene of each species [34]. Together, the immoderate sequence variations of the selected SSU-rRNA and ITS1 regions as well as the lack of data from the Paraleishmania group could impede the phylogenetic estimation to exhibit concordant relationships. Nevertheless, when cautiously considering the intra-species relationships within *L. siamensis*, the relatively high degree of genetic distance within species compared with other species complex in the genus *Leishmania* implied that lineages PG and TR of *L. siamensis* might not be a species complex. This analysis, on the other hand, strengthens the possibility that these two lineages might be of different species. Hence, further molecular studies on these two lineages using multilocus enzyme electrophoresis (MLEE) as the classical method/gold standard of *Leishmania* typing or MLST based on the protein genes used for MLEE would enhance the understanding of the phylogenetic basis of *L. siamensis*.

## Conclusion

The genetic analysis conducted in this study brings more insight into the phylogenetic relationships of *L. siamensis* covering intra- and interspecies aspects. Two *L. siamensis* lineages were proposed based on the findings from this study, of which lineage PG was the predominant one responsible for VL in Thailand. The existence of this lineage seems to be not restricted only to Thailand but also prevalent on other continents, causing the disease to affect livestock. Little is known whether the two *L. siamensis* lineages designated in this study have different parasite characteristics such as geographical distribution, virulence in humans, host preference, transmission vector, as well as drug sensitivity. Knowing their taxonomy precisely will shed further light on parasite biological properties linked to closely related species.

At present, more VL cases caused by *L. siamensis* have been increasingly detected in southern Thailand and have also spread widely in other regions of the country. The disease burden is significantly underestimated and the true incidence is not well reflected, as only a few published case reports are available. Further study is required for a large scale molecular epidemiological study of emerging VL disease caused by *L. siamensis* in Thailand.

## Consent

Written informed consent was obtained from the patient for publication of this report and any accompanying images.

## Competing interests

The authors declare that they have no competing interests.

## Authors’ contributions

SL participated in the study design, conceived the project, supervised the experiments, analyzed and interpreted the data, and co-wrote the manuscript. SS participated in the study design, performed the experiments, analyzed and interpreted the data, and co-wrote the manuscript. AH and HK performed the experiments. PT participated in the study design and conceived the project. PS and SO participated in specimen collection. MM participated in the study design, conceived the project, and co-wrote the manuscript. All authors read and approved the final manuscript.

## Supplementary Material

Additional file 1 **Sequence alignment of 348 bp of ITS1 region of *L. donovani*, *L. infantum*, *Leishmania *sp. (cow in Europe), *Leishmania *sp. (horse in Europe), *L. siamensis *(mare in the USA), *L. siamensis *lineage PG, and *L. siamensis *lineage TR.** Bases that are identical to those of the *L. siamensis* lineage PG are indicated by dots, missing bases are indicated by hyphens, and bases that are different from those of the *L. siamensis* lineage PG are given. Click here for file

Additional file 2 **Sequence alignment of 1380 bp of *hsp*70 region of *L. donovani*, *L. infantum*, *L. siamensis *lineage PG, and *L. siamensis *lineage TR.** Bases that are identical to those of the *L. siamensis* lineage PG are indicated by dots, missing bases are indicated by hyphens, and bases that are different from those of the *L. siamensis* lineage PG are given. Click here for file

Additional file 3 **Sequence alignment of 816 bp of *cyt *b region of *L. donovani*, *L. infantum*, *L. enrietti*, *L. siamensis *lineage PG, and *L. siamensis *lineage TR.** Bases that are identical to those of the *L. siamensis* lineage PG are indicated by dots, missing bases are indicated by hyphens, and bases that are different from those of the *L. siamensis* lineage PG are given. Click here for file
