# Supplementary figures and images for: Multilocus characterization and phylogenetic analysis of Leishmania siamensis isolated from autochthonous visceral leishmaniasis cases, southern Thailand
Source: BMC Microbiol. 2013 Mar 18;13:60. doi: 10.1186/1471-2180-13-60 (PMC3724499; doi:10.1186/1471-2180-13-60)

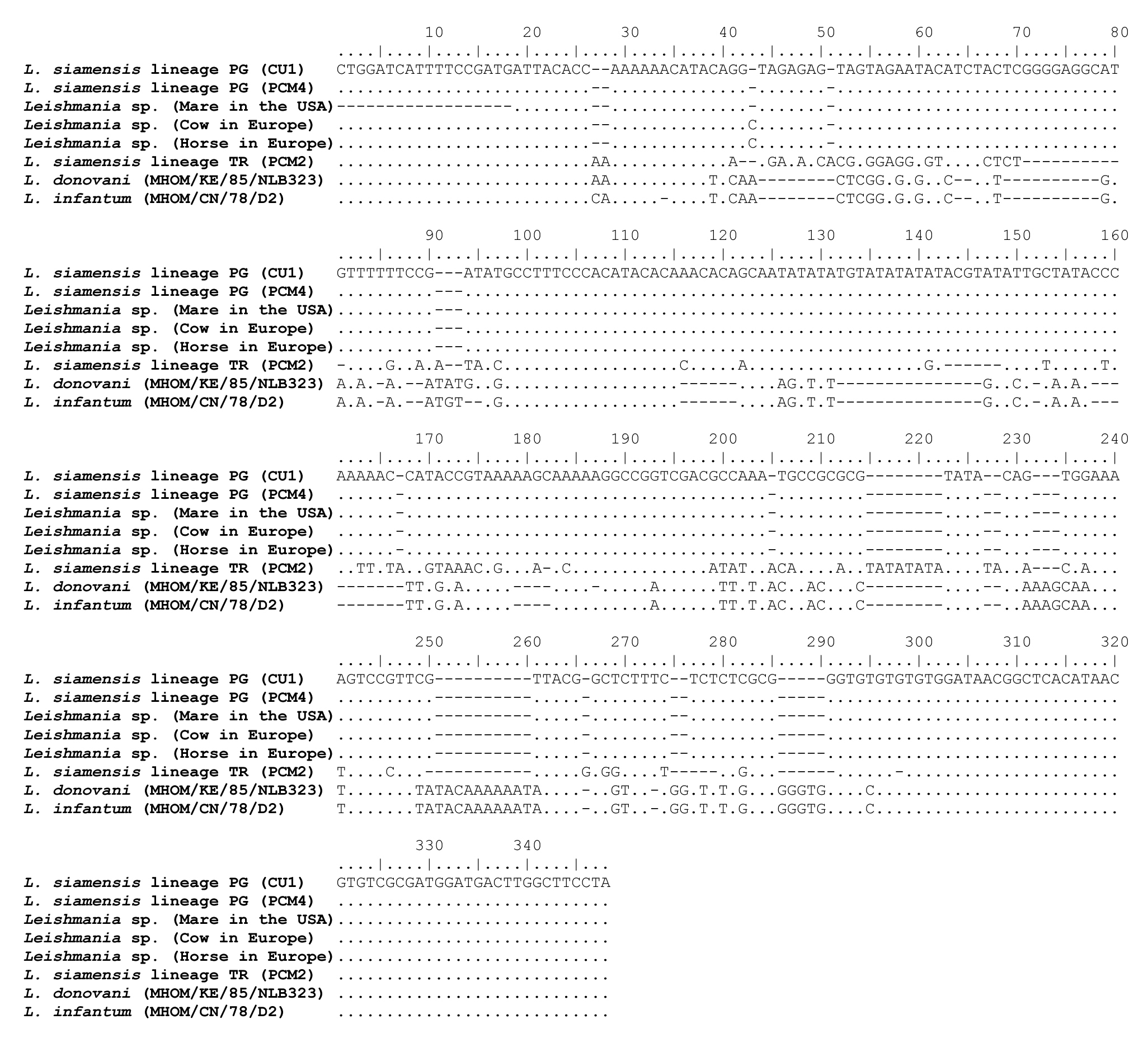

Supplement: Additional file 1 — Sequence alignment of 348 bp of ITS1 region of L. donovani, L. infantum, Leishmania sp. (cow in Europe), Leishmania sp. (horse in Europe), L. siamensis (mare in the USA), L. siamensis lineage PG, and L. siamensis lineage TR. Bases that are identical to those of the L. siamensis lineage PG are indicated by dots, missing bases are indicated by hyphens, and bases that are different from those of the L. siamensis lineage PG are given. [file 1471-2180-13-60-S1.jpeg]

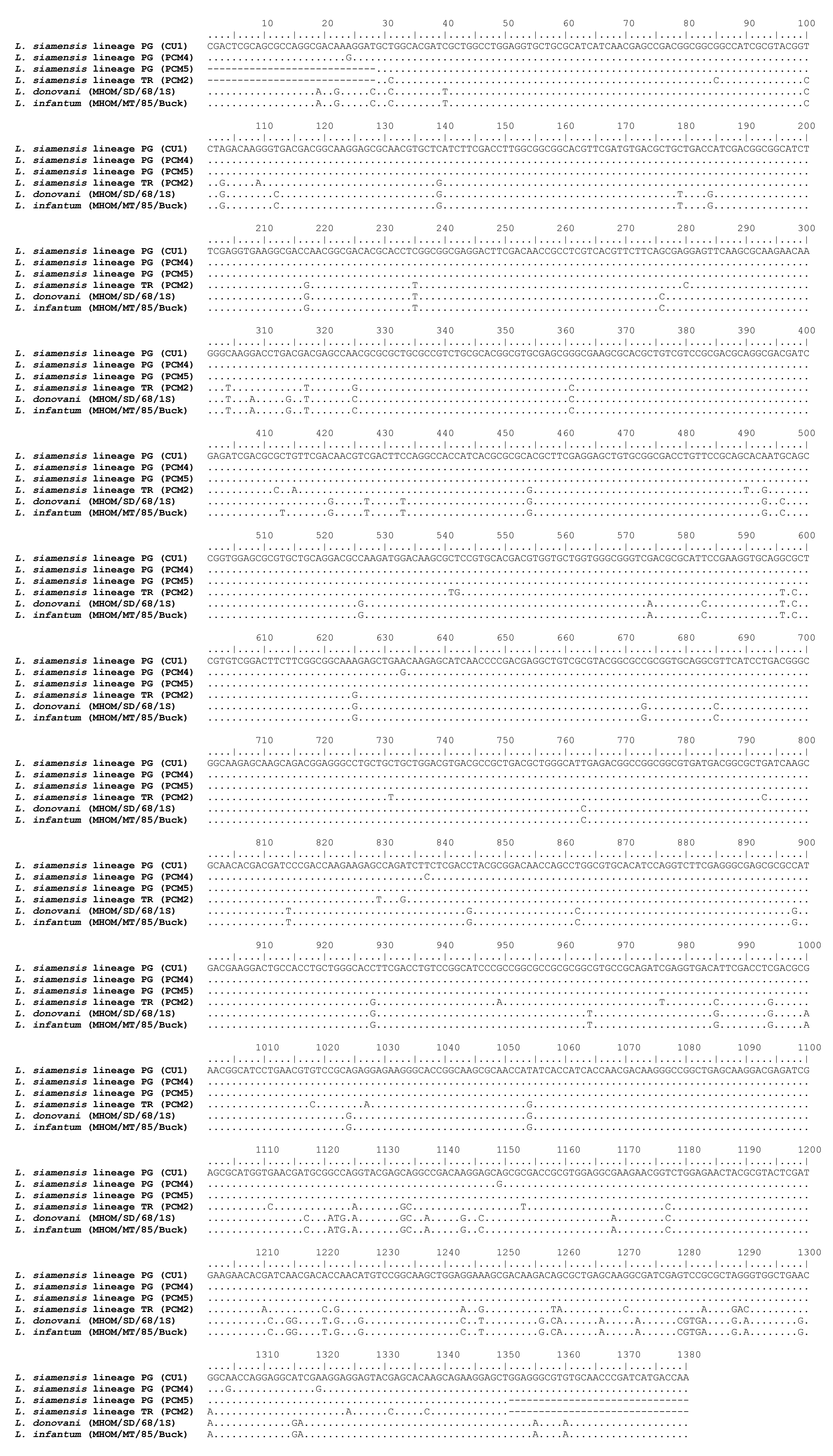

Supplement: Additional file 2 — Sequence alignment of 1380 bp of hsp70 region of L. donovani, L. infantum, L. siamensis lineage PG, and L. siamensis lineage TR. Bases that are identical to those of the L. siamensis lineage PG are indicated by dots, missing bases are indicated by hyphens, and bases that are different from those of the L. siamensis lineage PG are given. [file 1471-2180-13-60-S2.jpeg]

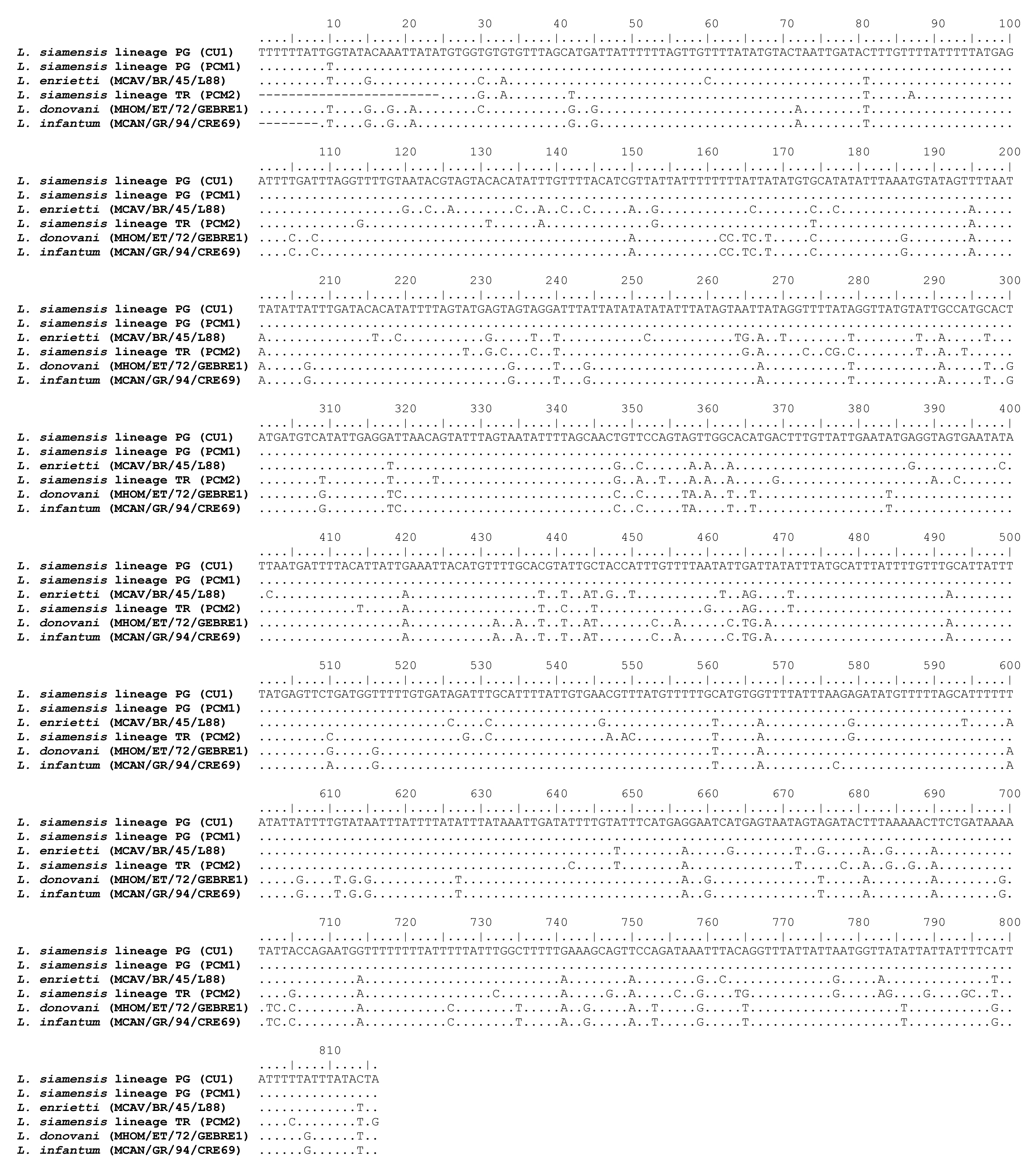

Supplement: Additional file 3 — Sequence alignment of 816 bp of cyt b region of L. donovani, L. infantum, L. enrietti, L. siamensis lineage PG, and L. siamensis lineage TR. Bases that are identical to those of the L. siamensis lineage PG are indicated by dots, missing bases are indicated by hyphens, and bases that are different from those of the L. siamensis lineage PG are given. [file 1471-2180-13-60-S3.jpeg]
